# Supplementary material for: Molecular basis of VEGFR1 autoinhibition at the plasma membrane
Source: Nat Commun. 2024 Feb 14;15:1346. doi: 10.1038/s41467-024-45499-2 (PMC10866885; doi:10.1038/s41467-024-45499-2)
Supplement: Supplementary file 1 — Supplementary file [file 41467_2024_45499_MOESM1_ESM.pdf]

Molecular basis of VEGFR1 autoinhibition at the plasma membrane

Manas Pratim Chakraborty[a], Diptatanu Das[a], Purav Mondal[a], Pragya Kaul[a], Soumi Bhattacharyya[a] Prosad Kumar Das[a] and Rahul Das[a],[b]*

[a] Department of Biological Sciences, Indian Institute of Science Education and Research Kolkata, Mohanpur campus, Mohanpur-741246, India

[b] Centre for Advanced Functional Materials, Indian Institute of Science Education and Research Kolkata, Mohanpur campus, Mohanpur-741246, India

Supporting Information

Corresponding authors

Rahul Das: rahul.das@iiserkol.ac.in

Supplementary Table 1: List of Antibodies

Antibodies	Source	Details	Dilution
VEGFR2 Rabbit monoclonal antibody	Cell Signaling Technology, Danvers, MA, USA	Cat # 2479S<br>Lot: 18	1:1000 (for IB)
Anti phosphotyrosine Antibody	Abcam<br>(Waltham, MA 02453, USA)	Cat # ab179530<br>Lot: GR198792-25	1:1000 (for IB)
Phospho-VEGF Receptor 2 (Tyr1175) Rabbit monoclonal antibody (19A10)	Cell Signaling Technology, (Danvers, MA, USA)	Cat # 2478T<br>Lot:15	1:200(for IF)<br>1:1000 (for IB)
VEGFR1 Goat polyclonal antibody	R & D system<br>(Minneapolis, MN, USA)	Cat # AF321<br>Lot: AHT2018011	1:1000 (for IB)
Phospho-VEGF Receptor 1 (Tyr1213) Rabbit polyclonal antibody	My BioSource<br>(San Diego, CA, USA)	Cat # MBS9600975<br>Lot: 07/2021	1:200(for IF)
Human Phospho-VEGFR1/Flt-1 (Y1213) Antibody	R & D system<br>(Minneapolis, MN, USA)	Cat # AF4170<br>Lot: ZKS0318061	1:200(for IF)<br>1:1000(for IB)
HA mouse monoclonal antibody	Biolegend<br>(San Diego, CA, USA)	Cat # 901501<br>Lot: B318172	1:2000 (for IB)
Mouse HRP Secondary antibody	Cell Signaling Technology, Danvers, MA, USA	Cat # 7076S<br>Lot: 33	1:3000 (for IB)
Rabbit HRP Secondary antibody	Abcam<br>(Waltham, MA 02453, USA)	Cat# 50095<br>Lot: 2960660	1:2000 (for IB)
Goat HRP Secondary antibody	Abcam<br>(Waltham, MA 02453, USA)	Cat #Ab6717<br>Lot: GR267728-27	1:2000 (for IB)
Rabbit FITC conjugated secondary antibody	Abcam<br>(Waltham, MA 02453, USA)	Cat # Ab6885<br>Lot: GR3391568-I	1:2000 (for IB)

Supplementary Table 2: List of constructs and primers:

Construct	Primer	Sequence
VEGFR2 -C482R	Fwd	GAC AAA CCC ATA CCC TTG TGA AGA ATG GAG AAG TG
	Rev	CACTTCTCCATTCTTCACAAGGGTATGGGTTTGTC
VEGFR1 -C472R	Fwd	GTT CTG GCA CCC CCG TAA CCA TAA TCA TTCC
	Rev	GGAATGATTATGGTTACGGGGGTGCCAGAAC
VEGFR1-TM^{VEGFR2}	TM_FP1	TACCGGACTCAGATCTCGAGATGGTCAGCTACTGGGACACCGG
	TM_RP1	AGAATAATGATTTCCAAGTTAGACTTGTCCGAGGTTCCTTGAACAG
	TM_FP2	AACTTGGAAATCATTATTCTAGTAGGCACGGC
	TM_RP2	AGACCTTTTCATTTTTCGTAGGATGATGACAAGAAGTAGC
	TM_FP3	CGAAAAATGAAAAGGTCTTCTTCTGAAATAAAGACTGACTACC
	TM_RP3	GGTGGCGACCGGTGGATCCACGATGGGTGGGGTGGAGTACAGGA
VEGFR1-JM^{VEGFR2}	JM_FP1	TACCGGACTCAGATCTCGAGATGGTCAGCTACTGGGACACCGG
	JM_RP1	TTGGCCCGCTTAACGGTCCGGATAAAGAGGGTTAATAGGAGCC
	JM_FP2	CGG ACC GTT AAG CGG GCC
	JM_RP2	CCG GTC TCT GGG GAA TTC C
	JM_FP3	GGGAATTCCCCAGAGACCGGCTTAAACTGGGCAAATCACTTG
	JM_RP3	GGTGGCGACCGGTGGATCCACGATGGGTGGGGTGGAGTACAGGA
VEGFR1-TMJM^{VEGFR2}	TMJM_FP1	TACCGGACTCAGATCTCGAGATGGTCAGCTACTGGGACACCGG
	TMJM_RP1	AGAATAATGATTTCCAAGTTAGACTTGTCCGAGGTTCCTTGAACAG
	TMJM_FP2	AACTTGGAAATCATTATTCTAGTAGGCACGGC
	TMJM_RP2	CCGGTCTCTGGGGAATTCCCATTTGC
	TMJM_FP3	GGGAATTCCCCAGAGACCGGCTTAAACTGGGCAAATCACTTGGAAGAGGGG
	TMJM_RP3	GGTGGCGACCGGTGGATCCACGATGGGTGGGGTGGAGTACAGGA
VEGFR1 ΔSSS	Fwd	CCCTCTTTATCCGAAAAATGAAAAGGGAAATAAAGACTGACTACCTATC
	Rev	GATAGGTAGTCAGTCTTTATTTCCCTTTTCATTTTTCGGATAAAGAGGG
VEGFR1-K1079Q	Fwd	GACAAAATCTACAGCACCCAGAGCGACGTGTGG
	Rev	CCACACGTCGCTCTGGGTGCTGTAGATTTTGTC

VEGFR1-R1142S	Fwd	CTGGCACAGAGACCCATCAGAAAGGCCAAGATTTGC
	Rev	GCAAATCTTGGCCTTTCTGATGGGTCTCTGTGCCAG
VEGFR1-R1146T	Fwd	CCATCAGAAAGGCCAACATTTGCAGAACTTGTGG
	Rev	CCACAAGTTCTGCAAATGTTGGCCTTTCTGATGG
VEGFR1 -D1022N	Fwd	GTG CAT TCA TCG GAA CCT GGC AGC GAG
	Rev	CTCGCTGCCAGGTTCCGATGAATGCAC
HA-VEGFR1	Fwd	CCTGAACTGAGTTTAAAAGGCACCC
	Rev	AGCGTAATCTGGAACATCGTATGGGTAATCTTTTAATTTTGAACCTGAACTAGATCC
HA-VEGFR1 ΔECD	Fwd	GTTCAAGGAACCTCGGACAAGTC
	Rev	AGCGTAATCTGGAACATCGTATGGGTAATCTTTTAATTTTGAACCTGAACTAGATCC
HA-VEGFR2 ΔECD	Fwd	GAAGGTGCCCAGGAAAAGACGAAC
	Rev	AGCCTTAATTGTAAGTATGTCTTTTTGTATGC
HA-VEGFR1 ΔECD -D1022N	Fwd	GTG CAT TCA TCG GAA CCT GGC AGC GAG
	Rev	CTCGCTGCCAGGTTCCGATGAATGCAC
HA-VEGFR1-TM^{VEGFR2} ΔECD	Fwd	GTG CAT TCA TCG GAA CCT GGC AGC GAG
	Rev	CTCGCTGCCAGGTTCCGATGAATGCAC
HA-VEGFR1-TMJM^{VEGFR2} ΔECD	Fwd	GTG CAT TCA TCG GAA CCT GGC AGC GAG
	Rev	CTCGCTGCCAGGTTCCGATGAATGCAC
HA-VEGFR1-JM^{VEGFR2} ΔECD	Fwd	GTG CAT TCA TCG GAA CCT GGC AGC GAG
	Rev	CTCGCTGCCAGGTTCCGATGAATGCAC
VEGFR1-TM^{gPA}- G83I	Fwd	GTGATGGCTATTGTTATTGGAACG
	Rev	CCCAAAAATAATGAGCTCCAG
VEGFR1-TM^{gPA}	gpA_FP1	TACCGGACTCAGATCTCGAGATGGTCAGCTACTGGGACACCGG
	gpA_RP1	CTCCAGATTAGACTTGTCCGAG
	gpA_FP2	CGGACAAGTCTAATCTGGAGCTCATTATTTTTGGGGTGATGG
	gpA_RP2	ACCGTAAGAAATTAAGAGGATCGTTCCAATAACACC
	gpA_FP3	TCCTCTTAATTTCTTACGGTATC CGA AAA ATG AAA AGG TCT TC
	gpA_RP3	GGTGGCGACCGGTGGATCCACGATGGGTGGGGTGGAGTACAGGA

T667V	Fwd	CTG GAG CTG ATC ATT CTA ACA TGC ACC
	Rev	GGT GCA TGT TAG AAT GAT CAG CTC CAG
T669I	Fwd	GAGCTGATCACTCTAGTATGCACCTGTGTGGC
	Rev	GCCACACAGGTGCATACTAGAGTGATCAGCTCC
C670G	Fwd	CTGATCACTCTAACAGGCACCTGTGTGGCTG
	Rev	CGCAGCCACACAGGTGCCTGTTAGAGTGATCAGC

Supplementary Table 3: Diffusion coefficient of different VEGFR1 and VEGFR2 constructs

Constructs	$D_{confocal}$ ($\mu m^2.s^{-1}$)	
	$-VEGF_{165}$	$+VEGF_{165}$
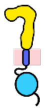 VEGFR1- wt	0.0385 ± 0.0179	0.0177 ± 0.0071
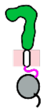 VEGFR2-wt	0.0206 ± 0.0075	0.0107 ± 0.0040
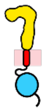 VEGFR1-TM^{GPA}	0.0211502 ± 0.0054	-
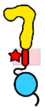 VEGFR1-TM$^{GPA(G83I)}$	0.0332 ± 0.0125	-
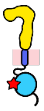 VEGFR1-D1022N	0.0325 ± 0.01509	0.01565 ± 0.0056
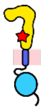 VEGFR1-C471R	0.0348 ± 0.0142	0.0172 ± 0.0071
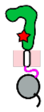 VEGFR2-C482R	0.0176 ± 0.0060	0.0125 ± 0.0025
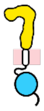 VEGFR1-TM^{VEGFR2}	0.022353 ± 0.0068	0.0129 ± 0.0032
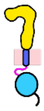 VEGFR1-JM^{VEGFR2}	0.0205 ± 0.0059	0.0127 ± 0.0047
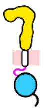 VEGFR1-TMJM^{VEGFR2}	0.0202 ± 0.0070	0.0131 ± 0.0050

Supplementary Table 4: Tyrosine Phosphorylation rate and phosphorylation half-life of VEGFR constructs after treatment with $VEGF_{165}$.

	Construct	Phosphorylation rate (arb. units/min)	Phosphorylation half-life (min)
		$+VEGF_{165}$	$VEGF_{165}$ mediated
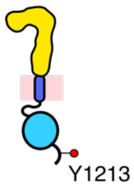	VEGFR1-wt	0.07 ± 0.01	14.3 ± 4.4
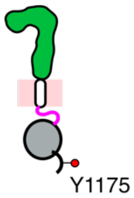	VEGFR2-wt	0.17 ± 0.02	> 60
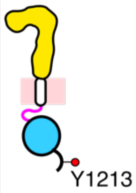	VEGFR1-TM-JM^{VEGFR2}	0.16 ± 0.03	48.6 ± 13.8

Supplementary Table 5: RMSDs of VEGFR1 model structures with respect to VEGFR1 crystal structure (PDB ID: 3HNG).

Top 5 Models of VEGFR1	Backbone RMSD wrt. VEGFR1 (PDB ID: 3HNG) (in Å)
Model 1	0.589
Model 2	0.617
Model 3	0.569
Model 4	0.599
Model 5	0.822

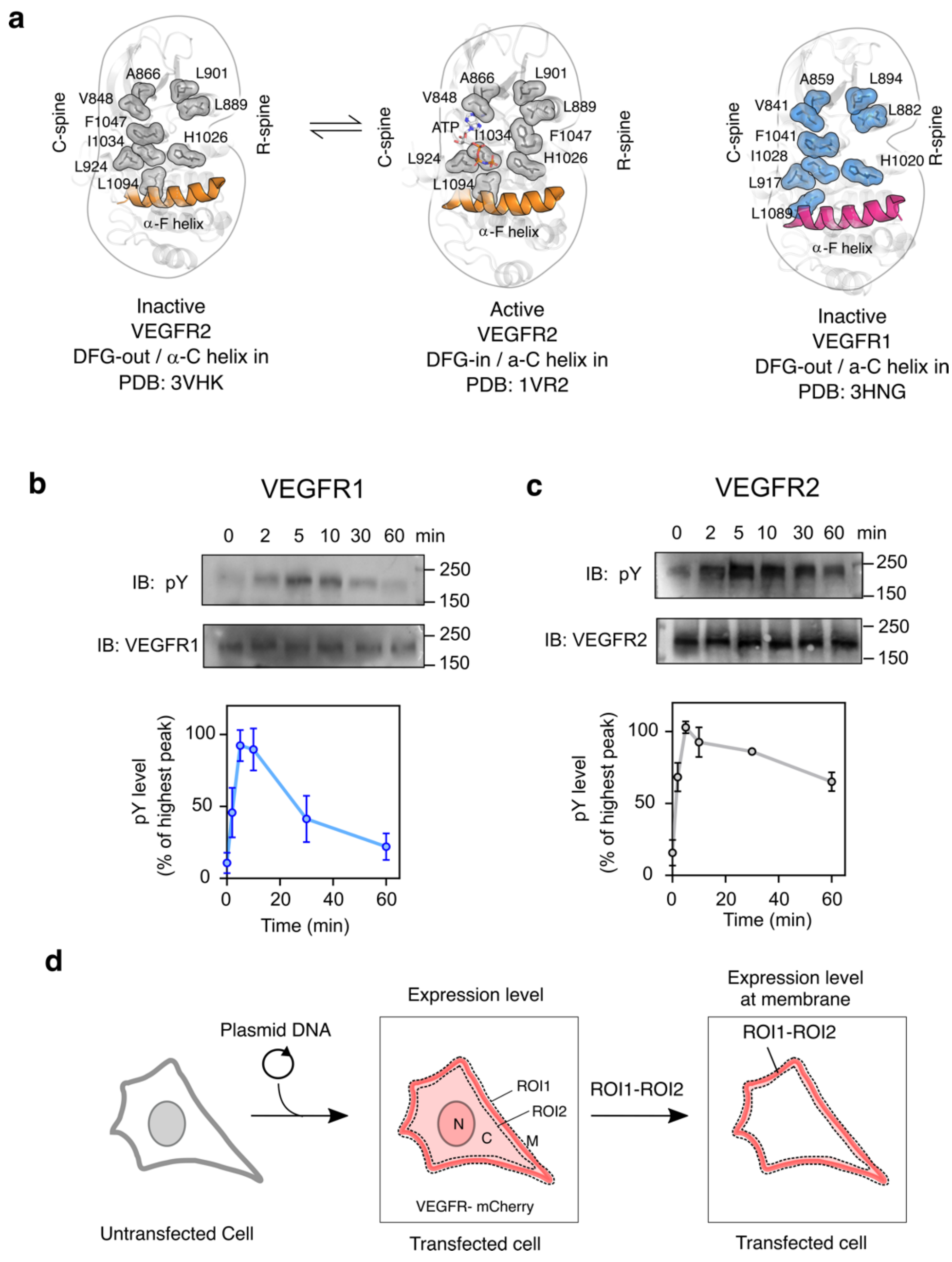

Supplementary Figure 1: Total phosphorylation of VEGFR1 in comparison to VEGFR2 in CHO cell line.

(a) Structural analysis of conserved signature motifs (Regulatory and Catalytic spine) in the indicated VEGFR2 (Inactive, PDB: 3VHK[1] and active, PDB: 1VR2[2]) and VEGFR1 kinase domain crystal structures.

(b-c) In the upper panel, the immunoblot shows the total phosphorylation level of VEGFR1 or VEGFR2 at the indicated time points after activating the transfected CHO cell line with 50 nM $VEGF_{165}$. The lower panel is the plot of the phosphorylation level of total tyrosine residue as a function of time. The phosphorylation level is analyzed from the densitometric measurement of the immunoblots. The t1/2 is determined by fitting the decay of the highest intensity observed to exponential decay. Data are presented as mean values ± SD from three independent experiments.

(d) Schematic representation of the single-cell assay of VEGFR activation. The area covered by ROI1 and ROI2 is shown by dotted lines. The membrane fraction is obtained by subtracting ROI2 from ROI1.

The space filled model was generated by using PyMOL Molecular Graphics System, Version 2.5.2 Schrödinger, LLC. The plots were generated by GraphPad Prism Ver 9.5.1. The schematics were drawn using Inkscape Ver 1.2. Source data are provided as a Source Data file for panels b-c.

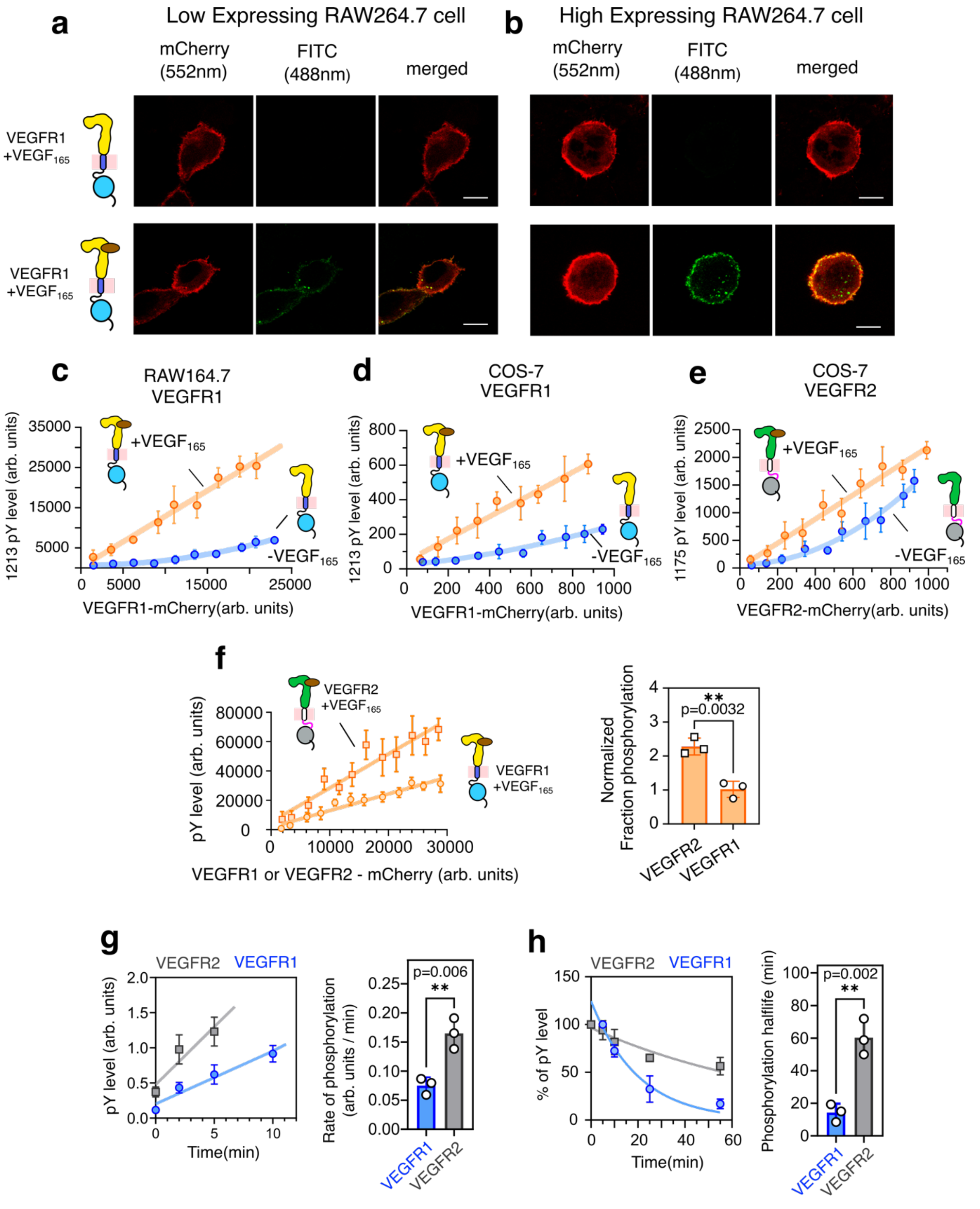

Supplementary Figure 2: Single-cell analysis of VEGFR activation and tyrosine phosphorylation kinetics

(a-b) Confocal images of VEGFR1 fused to mCherry and Y1213 phosphorylation level (in green) were measured in a transiently RAW264.7 cell line. Panels a and b show the phosphorylation level of Y1213 in low or high expressing VEGFR1, respectively, with and without ligand treatment. Scale bar = 5 µm

(c-d) The Y1213 phosphorylation versus VEGFR1 expression levels in RAW264.7 and COS-7 cell lines are plotted. Individual data points represent the mean expression and phosphorylation level for the selected cell with comparable expression levels (2500 arb. units). In panel c, n= 77 (VEGFR1-VEGF$_{165}$), 106 (VEGFR1+VEGF$_{165}$), and in panel d, n= 86 (VEGFR1-VEGF$_{165}$), 116 (VEGFR1+VEGF$_{165}$) cells were examined over seven independent experiments. Data are presented as mean values ± SD

(e) The Y1175 phosphorylation is plotted against the expression level of VEGFR2 transiently expressed in the COS-7 cell line. n= 125 (VEGFR2-$VEGF_{165}$), 145 (VEGFR2+$VEGF_{165}$), cells were examined over five independent experiments. Data are presented as mean values ± SD.

(f) The left panel compares the ligand-dependent phosphorylation levels of VEGFR1 and VEGFR2 as a function of their expression levels. The bar plot at the right depicts the fraction of VEGFR1 or VEGFR2 phosphorylation, as determined from the slope of the curve fitted to a linear equation. Data are presented as mean values ± SD from three independent experiments. An unpaired two-tailed t-test was used to calculate significance.

(g) The left panel shows the fitting of the densitometric analysis of Y1175 (grey) and Y1213 (blue) phosphorylation as a function of time in Figure 2g to a linear equation. The bar plot at right shows the rate of phosphorylation as determined by the slope. Data are presented as mean values ± SD from three independent experiments. An unpaired two-tailed t-test was used to calculate significance.

(h) The left panel plots the densitometric analysis of Y1175 (gray) and Y1213 (blue) dephosphorylation as a function of time in Figure 2g to a linear equation. The bar plot shows the dephosphorylation rate as determined by fitting the data points to the exponential decay curve (left panel). Data are presented as mean values ± SD from three independent experiments. An unpaired two-tailed t-test was used to calculate significance.

All data were plotted using GraphPad Prism Ver 9.5.1. The confocal images were generated using Fiji Ver 1.54f [3]. Data analyses were performed using GraphPad Prism version 9.5.1. All the schematics and icons were designed using Inkscape Ver1.2. Source data are provided as a Source Data file for panels c-h.

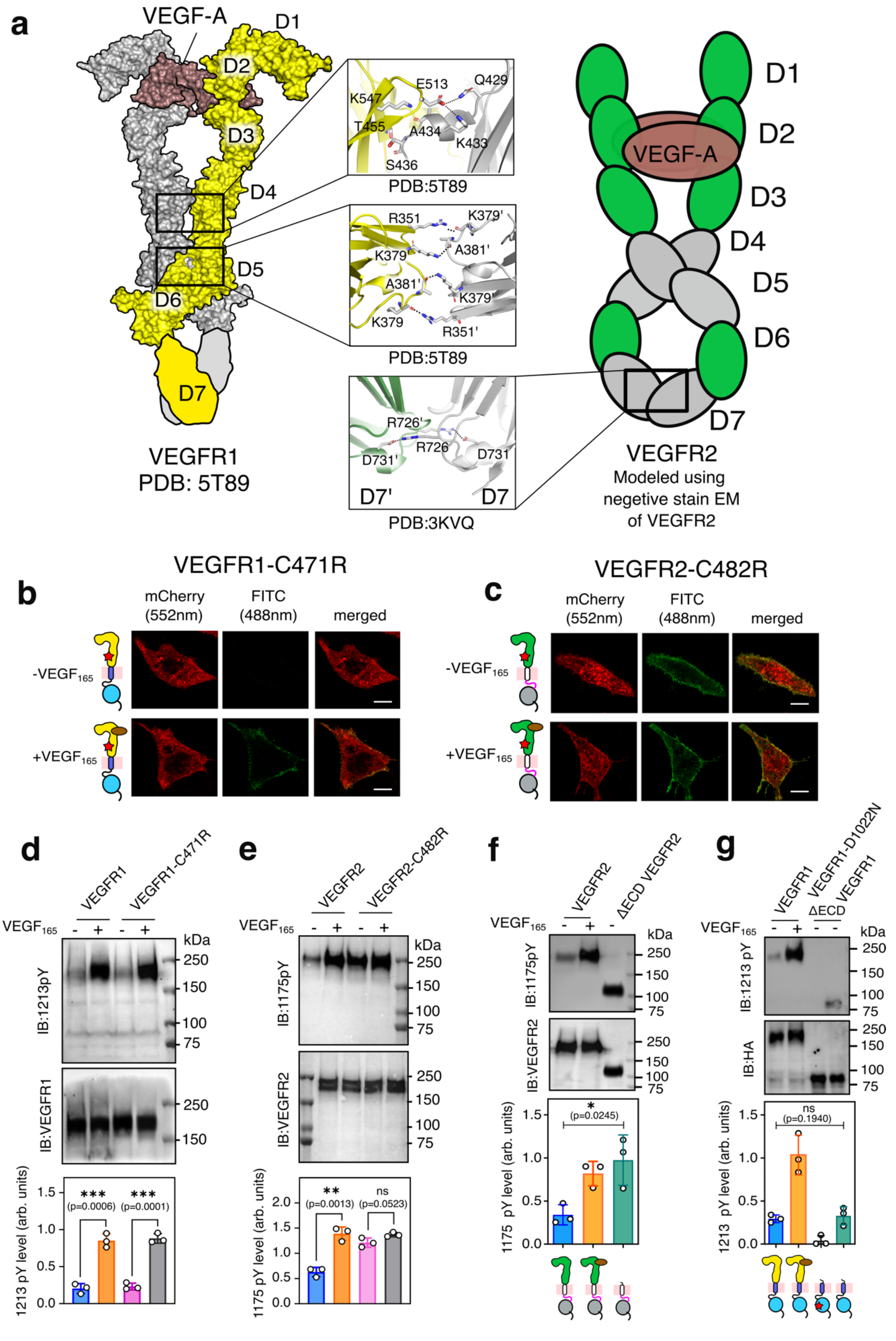

Supplementary Figure 3: Function of ECD and constitutively active mutant in ligand-dependent VEGFR activation

(a) The left panel shows the VEGFR1(D1-D6) structure. The D7 is modeled using VEGFR2-D7 as a template[4]. The right panel shows a schematic model of VEGFR2 (D1-D7) from a negative stain electron

micrograph[5]. The middle panel shows the cross-section of the dimerization arms (D4, D5, and D7). The key residues at the dimer interface are labeled.

(b-c) Confocal images showing the phosphorylation level of mCherry fused VEGFR1-C471R (b) or VEGFR2-C482R (c) in transiently transfected CHO cell lines.

(d) In the upper panel, immunoblot shows the Y1213 phosphorylation of VEGFR1-C471R mutant in the presence and absence of ligand. Bar plots in the lower panel represent the densitometric quantitation of the Y1213 phosphorylation level. Data are presented as mean values ± SD from three independent experiments. An unpaired two-tailed t-test was used to calculate significance.

(e) In the upper panel, immunoblot shows the Y1175 phosphorylation of constitutively activating VEGFR2-C482R mutant in the presence and absence of ligand. Bar plots, in the lower panel, represent the densitometric quantitation of the Y1175 phosphorylation level. Data are presented as mean values ± SD from three independent experiments. An unpaired two-tailed t-test was used to calculate significance.

(f-g) The immunoblots show the effect of ECD deletion (Δ ECD) on the phosphorylation of Y1175 in VEGFR2 (panel f) and Y1213 in VEGFR1 (panel g). The densitometric analysis of the Western blot is shown below in each panel. The phosphorylation level for each construct is normalized against the respective receptor expression level. In panel g, VEGR1-D1022N represents a kinase-dead mutant. The concentration of the VEGFR1 is determined using an antiHA antibody. Data are presented as mean values ± SD from three independent experiments. An unpaired two-tailed t-test was used to calculate significance.

The space filled model was generated by PyMOL Molecular Graphics System, Version 2.5.2 Schrödinger, LLC. The confocal images were generated using Fiji Ver 1.54f [3]. The plots were generated by GraphPad Prism Ver 9.5.1. Data analyses were performed using GraphPad Prism version 9.5.1. All the schematics and icons were designed using Inkscape 1.2. Source data are provided as a Source Data file for panels d-g.

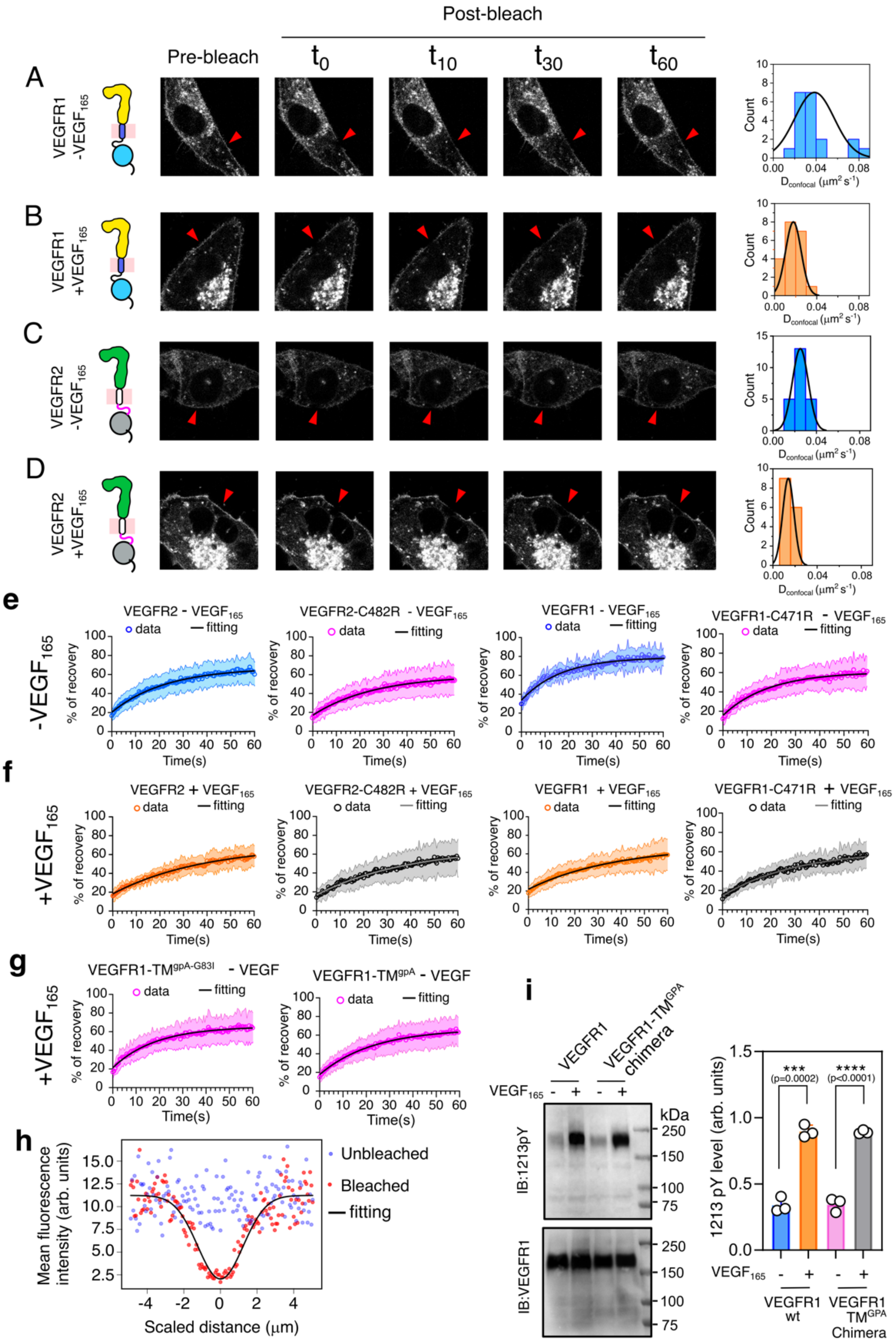

Supplementary Figure 4: Analysis of VEGFR dimer by FRAP

(a-d) Time-lapse image of CHO cell line transiently expressing indicated constructs of VEGFR. FRAP was performed on the plasma membrane of VEGFR1 (panels a, and b) or VEGFR2 (panels c, and d) transfected CHO cell line in the presence (panels b, and d) or absence (panels a, and c) of $VEGF_{165}$. A red arrow indicates the bleach spot. Recovery of fluorescence is shown at indicated time points. The right panel shows the normal distribution of diffusion coefficients.

(e-f) FRAP profile of indicated constructs in the absence (e) or presence (f) $VEGF_{165}$, each profile is fitted to the first order exponential equation. n = 25 ($VEGFR2-VEGF_{165}$), 21 ($C482R-VEGF_{165}$), 20

(VEGFR1-$VEGF_{165}$), 24 (C471R-$VEGF_{165}$), 18 (VEGFR2+$VEGF_{165}$), 18 (C482R+$VEGF_{165}$) 20 (VEGFR1+$VEGF_{165}$), and 18 (C471R+$VEGF_{165}$) cells examined over eight independent experiments.

(g) FRAP profile of monomer (left panel) and dimer(right panel) control, as explained in Figure 3, in the absence of $VEGF_{165}$. n = 18 (VEGFR1-$TM^{gPA-G83I}$), 24 (VEGFR1-TM^{gPA}) cells examined over eight independent experiments.

(h) Determination of effective bleach spot radius(r_e) from post-bleach intensity profile.

(i) Immunoblot (left panel) showing the Y1213 phosphorylation level of VEGFR1-TM^{GPA} (TM dimer control) as compared to its wild-type control. Bar plots in the right panel represent the quantitation of the Y1213 phosphorylation level. Data are presented as mean values ± SD from three independent experiments.

FRAP analysis was done using Fiji Ver 1.54f [3]. The confocal images were generated using Fiji Ver 1.54f. The plots were generated by GraphPad Prism Ver 9.5.1. Data analyses were performed using GraphPad Prism version 9.5.1. All the schematics and icons were designed using Inkscape 1.2. Source data are provided as a Source Data file for panels e-i.

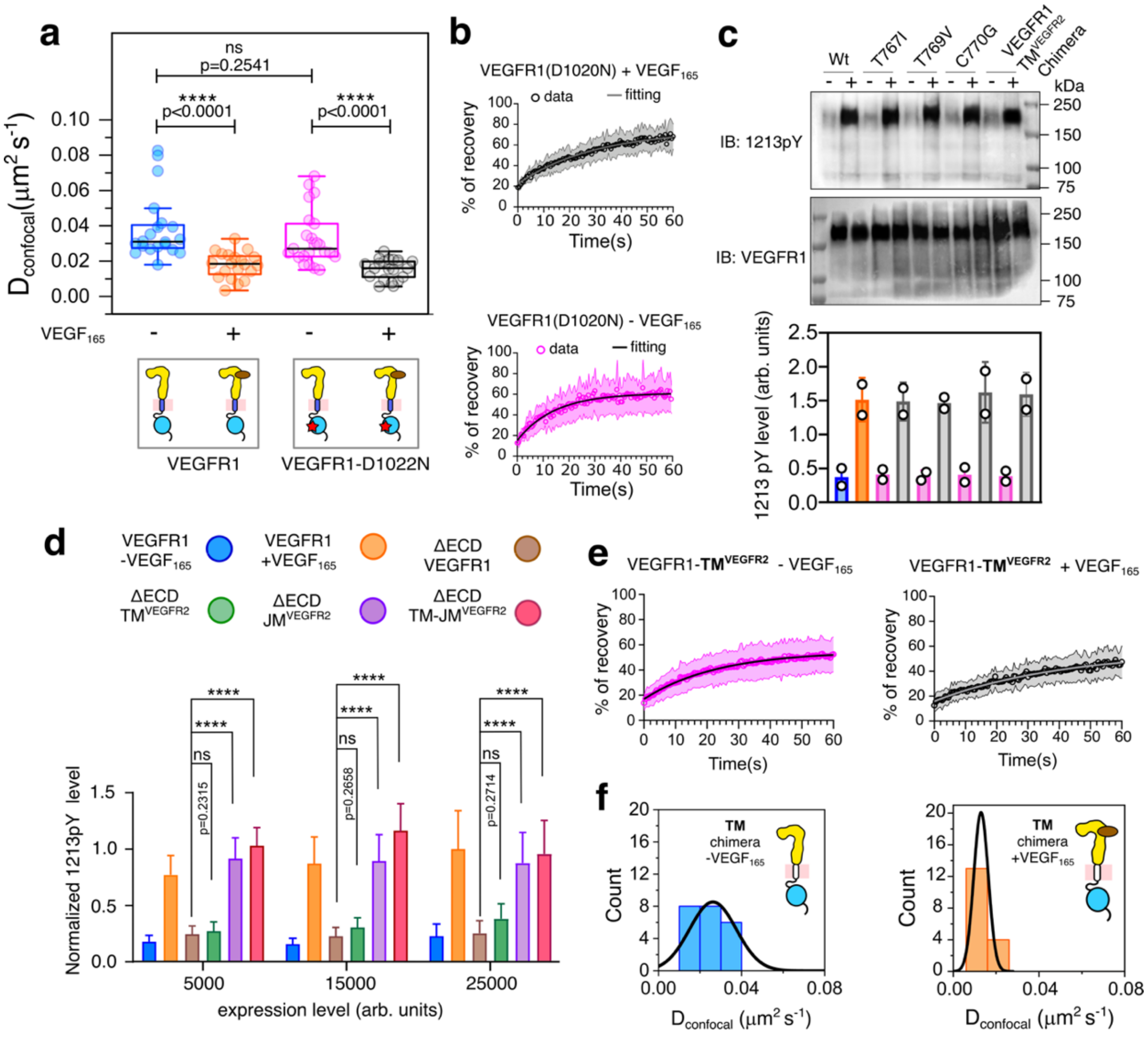

Supplementary Figure 5: Study of TM segment in activating VEGFR1

(a) Box plot of the diffusion coefficient of VEGFR1 and kinase-dead mutant (D1022N) determined from FRAP experiment. Each data point in the box plot reflects the diffusion coefficient of the selected cell, while the black line indicates the mean value. n=20 (VEGFR1-VEGF$_{165}$), 20 (VEGFR1+VEGF$_{165}$) 22 (D1022N-VEGF$_{165}$), and 24 (D1022N+VEGF$_{165}$) cells examined over eight independent experiments. An unpaired two-tailed t-test was used to calculate significance. Boxplots represent quartiles. The data points outside the whisker range are set as outliers. The black line inside the box represents the median value.

(b) FRAP profile of VEGFR1 kinase-dead mutant (D1022N) in the presence (left panel) and absence (right panel) of VEGF$_{165}$.

(c) Top panel is the Immunoblot of Y1213 phosphorylation in transmembrane domain mutants and chimeric constructs of VEGFR1. The densitometric quantification of Y1213Y phosphorylation is depicted by bar graphs (bottom panel).Data are presented as mean values ± SD from two independent experiments.

(d) The bar plots representing the normalized Y1213 phosphorylation level of VEGFR1-ECD deleted constructs, shown in Figure 4c. Data are presented as mean values ± SD from four independent experiments. An unpaired two-tailed t-test was used to calculate significance, ****$P < 0.0001$.

(e) FRAP profile of VEGFR1 -TM^{VEGFR2} chimera in the presence and absence of VEGF$_{165}$.

(f) Normal distribution of diffusion coefficient measured for VEGFR1 -TM^{VEGFR2} chimera in the presence and absence of ligand.

All the data were plotted using GraphPad Prism Ver 9.5.1. FRAP analysis was done using Fiji Ver 1.54f [3], and the boxplots were generated using Origin Pro 2020b. The immunoblot images were generated and analyzed using ImageJ Ver 1.53e. All the schematics and icons were designed using Inkscape Ver1.2. Source data are provided as a Source Data file for panels a-f.

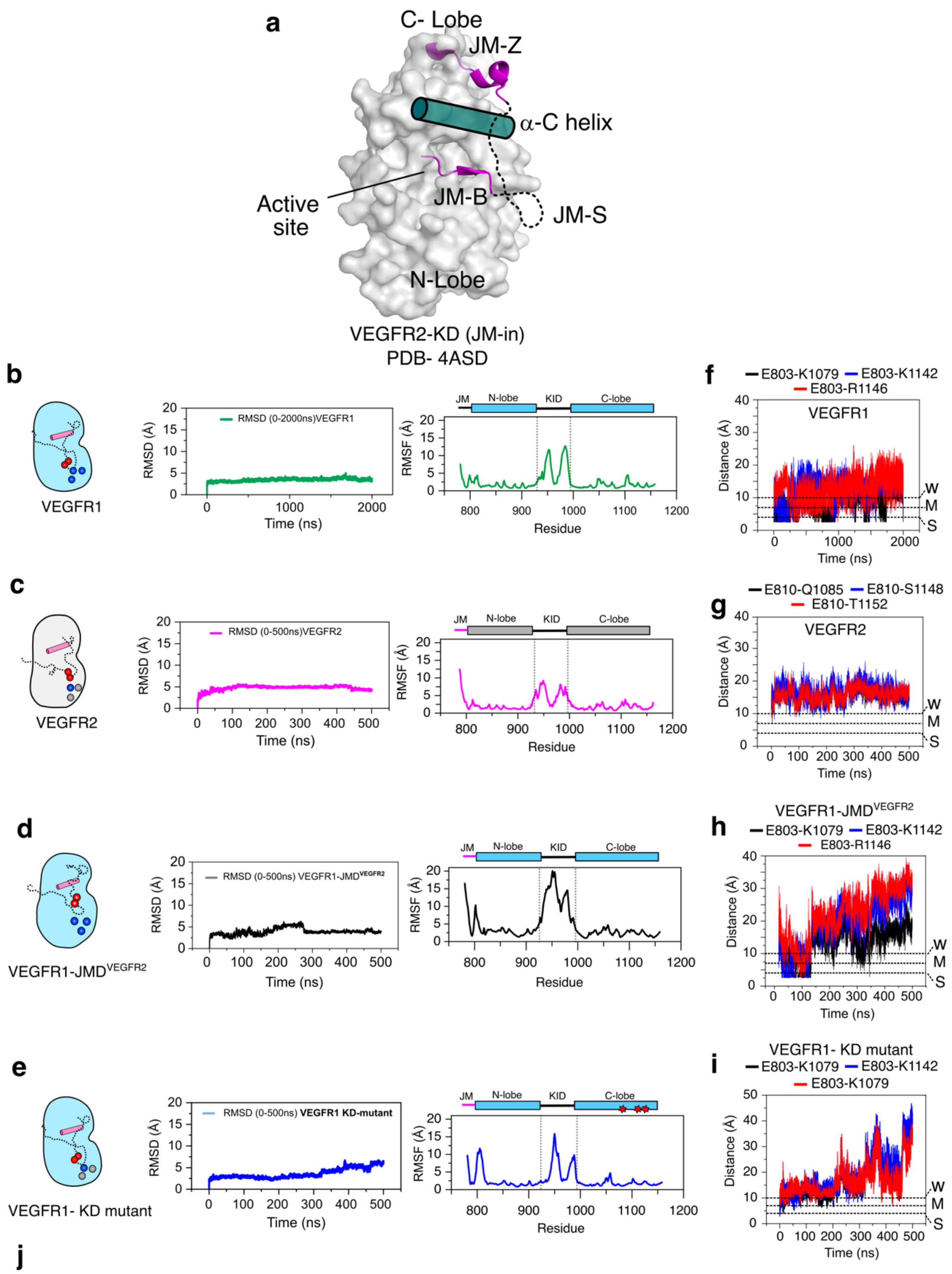

j

System Name	Simulation box dimensions (Protein edge to box edge distance)	Total number of atoms	Total number of water (TIP3P) molecules	Salt (NaCl) concentration	Lipid composition
VEGFR1-wt	10 Å	22,694	22,187	0.15M	NA
VEGFR2-wt	10 Å	19,624	19,137	0.15M	NA
VEGFR1-JMD^{VEGFR2}	10 Å	15,711	15,245	0.15M	NA
VEGFR1-KD mutant	10 Å	21,011	20,511	0.15M	NA

Supplementary Figure 6: Molecular dynamics simulation of JM-KD of VEGFR

(a) The inactive structure of VEGFR2 in JM-in conformation is shown as a space-filled model. The JM-S segment (in dotted lines) is modeled based on the PDGFR crystal structure (PDB ID: 5K5X)[6].

(b-e) The root mean square deviation (RMSD) and root mean square fluctuation (RMSF) for the indicated JM-KD constructs of VEGFR.

(f-i) The pairwise interatomic distances determined for the residues in the electrostatic latch are plotted against time for the constructs described in Figure 5 b-e. The horizontal lines represent the distance cut-off for the weak, medium, and strong electrostatic interactions[7,8].

(j) Table describing the details of the MD simulation setup.

The space filled model was generated by The PyMOL Molecular Graphics System, Version 2.5.2 Schrödinger, LLC. Plots were generated using Origin Pro 2020b. All the schematics and icons were designed using Inkscape Ver1.2. Source data are provided as a Source Data file for panels b-i.

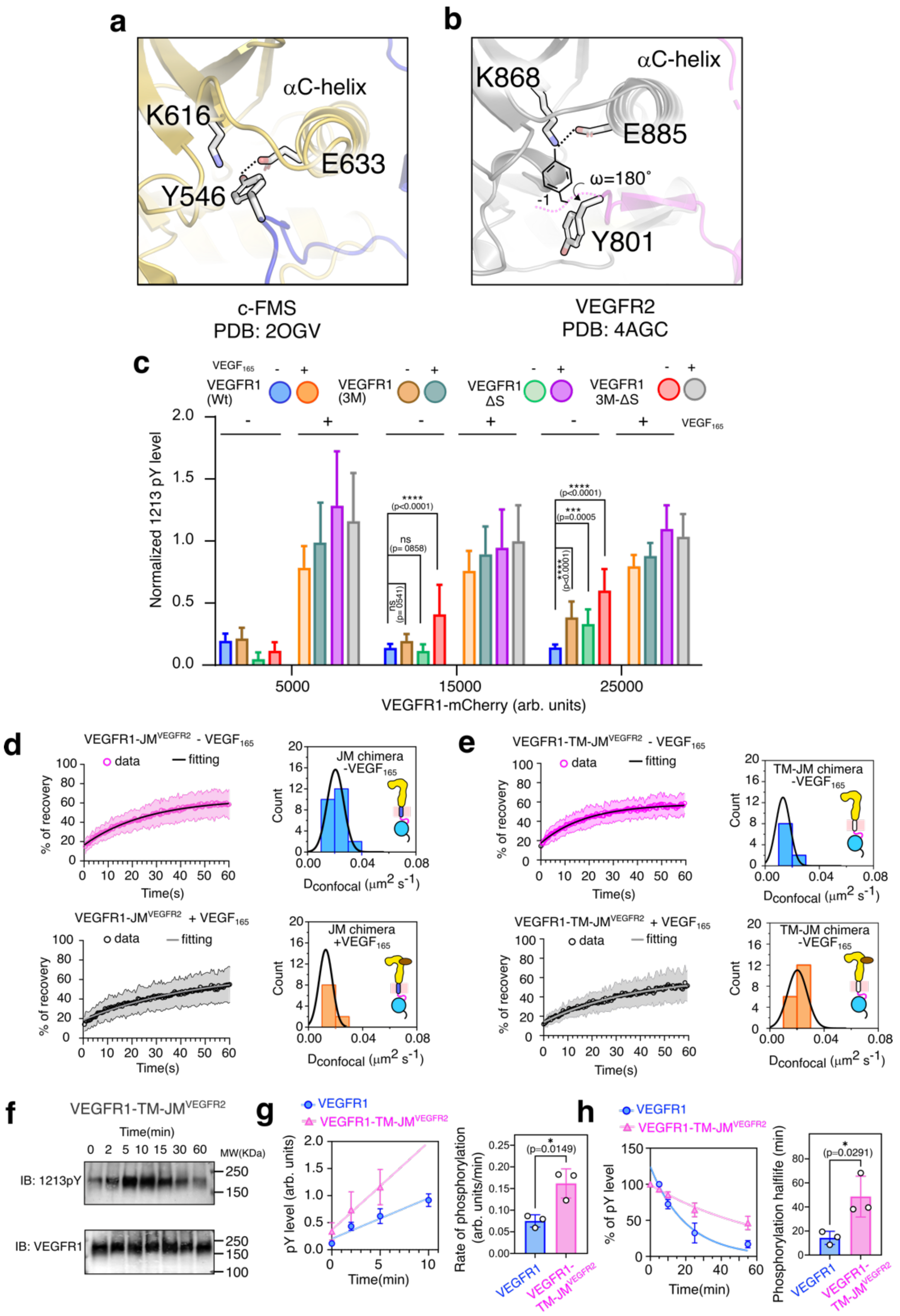

Supplementary Figure 7: Study of JM inhibition

(a) A close-up view of the inhibitory interaction between the Tyr546 and Glu633 in the JM-B and C-helix of the cFMS KD crystal structure (PDB ID: 2OGV)[9].

(b) Close-up view of the autoinhibitory conformation of the JM-B segment in the VEGFR2 crystal structure (PDB ID: 4AGC)[10]. The orientation of the conserved Y801 residue in the JM-B is shown. The

expected orientation of the Y801 residue is drawn considering the Y801 residue is moved to the -1 position, and the ω angle is rotated by 180°.

(c) The bar plot represents the normalized phosphorylation levels of different chimeric constructs with their respective receptor expression level corresponding to Figure 6d. Data are presented as mean values ± SD from six independent experiments. An unpaired two-tailed t-test was used to calculate significance.

(d-e) The left panel shows the FRAP profile of VEGFR1 -JM^{VEGFR2} and VEGFR1 -TM-JM^{VEGFR2} chimeras, respectively, in the presence and absence of ligand. The solid line represents the fitting to a first-order exponential equation. The right panel represents the normal distribution of the diffusion coefficient of indicated constructs. n=23 (VEGFR1-JM^{VEGFR2} -VEGF$_{165}$), 18 (VEGFR1-JM^{VEGFR2} +VEGF$_{165}$) 20 (VEGFR1-TMJM^{VEGFR2} - VEGF$_{165}$) and 17 (VEGFR1-TMJM^{VEGFR2} + VEGF$_{165}$) cells examined over eight independent experiments.

(f) The representative immunoblot of ligand-dependent Y1213 phosphorylation of VEGFR1-TM-JM^{VEGFR2} chimera measured over indicated time points.

(g) The rate of Y1213 phosphorylation is determined from the slope of the phosphorylation level vs time plot, measured from the densitometric analysis of Figure 6d. The phosphorylation rate is determined from the linear fit of phosphorylation at time t_0 to the highest phosphorylation level measured. The right panel shows the average phosphorylation rates measured from three independent experiments. Data are presented as mean values ± SD. An unpaired two-tailed t-test was used to calculate significance.

(h) The half-life of the phosphotyrosine residue Y1213 of VEGFR1 is determined from the exponential fitting of the phosphorylation decay from the highest phosphorylation observed in Figure 6d. The right panel shows the average phosphorylation half-life measured from three independent experiments. Data are presented as mean values ± SD. An unpaired two-tailed t-test was used to calculate significance.

The cartoon model was generated by The PyMOL Molecular Graphics System, Version 2.5.2 Schrödinger, LLC. FRAP analysis was done using Fiji Ver 1.54f [3]. The plots were generated by GraphPad Prism Ver 9.5.1. Data analyses were performed using GraphPad Prism version 9.5.1. All the schematics and icons were designed using Inkscape 1.2. Source data are provided as a Source Data file for panels c-h.

Supplementary References

1 Iwata, H. *et al.* A Back-to-Front Fragment-Based Drug Design Search Strategy Targeting the DFG-Out Pocket of Protein Tyrosine Kinases. *ACS Med Chem Lett* **3**, 342-346, doi:10.1021/ml3000403 (2012).

2 McTigue, M. A. *et al.* Crystal structure of the kinase domain of human vascular endothelial growth factor receptor 2: a key enzyme in angiogenesis. *Structure* **7**, 319-330, doi:10.1016/s0969-2126(99)80042-2 (1999).

3 Schindelin, J. *et al.* Fiji: an open-source platform for biological-image analysis. *Nat Methods* **9**, 676-682, doi:10.1038/nmeth.2019 (2012).

4 Markovic-Mueller, S. *et al.* Structure of the Full-length VEGFR-1 Extracellular Domain in Complex with VEGF-A. *Structure* **25**, 341-352, doi:10.1016/j.str.2016.12.012 (2017).

5 Ruch, C., Skiniotis, G., Steinmetz, M. O., Walz, T. & Ballmer-Hofer, K. Structure of a VEGF-VEGF receptor complex determined by electron microscopy. *Nat Struct Mol Biol* **14**, 249-250, doi:10.1038/nsmb1202 (2007).

6 Liang, L., Yan, X. E., Yin, Y. & Yun, C. H. Structural and biochemical studies of the PDGFRA kinase domain. *Biochem Biophys Res Commun* **477**, 667-672, doi:10.1016/j.bbrc.2016.06.117 (2016).

7 Weinkam, P., Pletneva, E. V., Gray, H. B., Winkler, J. R. & Wolynes, P. G. Electrostatic effects on funneled landscapes and structural diversity in denatured protein ensembles. *Proc Natl Acad Sci U S A* **106**, 1796-1801, doi:10.1073/pnas.0813120106 (2009).

8 Piana, S. *et al.* Evaluating the effects of cutoffs and treatment of long-range electrostatics in protein folding simulations. *PLoS One* **7**, e39918, doi:10.1371/journal.pone.0039918 (2012).

9 Walter, M. *et al.* The 2.7 A crystal structure of the autoinhibited human c-Fms kinase domain. *J Mol Biol* **367**, 839-847, doi:10.1016/j.jmb.2007.01.036 (2007).

10 McTigue, M. *et al.* Molecular conformations, interactions, and properties associated with drug efficiency and clinical performance among VEGFR TK inhibitors. *Proc Natl Acad Sci U S A* **109**, 18281-18289, doi:10.1073/pnas.1207759109 (2012).
