# Supplementary material for: Molecular basis of VEGFR1 autoinhibition at the plasma membrane
Source: Nat Commun. 2024 Feb 14;15:1346. doi: 10.1038/s41467-024-45499-2 (PMC10866885; doi:10.1038/s41467-024-45499-2)
Supplement: Supplementary file 3 — Reporting Summary [file 41467_2024_45499_MOESM3_ESM.pdf]

nature portfolio

Corresponding author(s): Rahul Das

Last updated by author(s): 14 December 2023

Reporting Summary

Nature Portfolio wishes to improve the reproducibility of the work that we publish. This form provides structure for consistency and transparency in reporting. For further information on Nature Portfolio policies, see our Editorial Policies and the Editorial Policy Checklist.

Statistics

For all statistical analyses, confirm that the following items are present in the figure legend, table legend, main text, or Methods section.

n/a	Confirmed	
☐	☒	The exact sample size (*n*) for each experimental group/condition, given as a discrete number and unit of measurement
☐	☒	A statement on whether measurements were taken from distinct samples or whether the same sample was measured repeatedly
☐	☒	The statistical test(s) used AND whether they are one- or two-sided<br>*Only common tests should be described solely by name; describe more complex techniques in the Methods section.*
☒	☐	A description of all covariates tested
☒	☐	A description of any assumptions or corrections, such as tests of normality and adjustment for multiple comparisons
☐	☒	A full description of the statistical parameters including central tendency (e.g. means) or other basic estimates (e.g. regression coefficient) AND variation (e.g. standard deviation) or associated estimates of uncertainty (e.g. confidence intervals)
☒	☐	For null hypothesis testing, the test statistic (e.g. *F*, *t*, *r*) with confidence intervals, effect sizes, degrees of freedom and *P* value noted<br>*Give P values as exact values whenever suitable.*
☒	☐	For Bayesian analysis, information on the choice of priors and Markov chain Monte Carlo settings
☒	☐	For hierarchical and complex designs, identification of the appropriate level for tests and full reporting of outcomes
☒	☐	Estimates of effect sizes (e.g. Cohen's *d*, Pearson's *r*), indicating how they were calculated

Our web collection on statistics for biologists contains articles on many of the points above.

Software and code

Policy information about availability of computer code

Data collection

Softwares used in this study are given below,
Immunoblots: Genesys
Confocal imaging: Leica Application Suite X (LASX)
GROMACS 2019.6
Measuring ROS generation:with Gen5

Data analysis

Softwares used in this study are given below,
Graphpad Prism 9.5.1
Origin Pro 2020b
Microsoft Office Excel 2019
Pymol 2.5.2
ImageJ win64
GROMACS 2019.6

The effective bleached spot radius in Figure S4H was determined by using the following script:

```
#!/usr/bin/env python3
import csv
import matplotlib.pyplot as plt
```

```
from matplotlib import rc
from scipy.optimize import curve_fit
import numpy as np

# Opening csv file and creating x,y list
dataset = open('FRAP1.csv','r')
csvreader = csv.reader(dataset)
rows = []
for row in csvreader:
 rows.append(row)

#Removing header files rows[0]
rows.pop(0)

#Creating x,y datapoints from elements of rows
x = np.array([float(rows[i][0]) for i in range(len(rows))])
y = np.array([float(rows[j][1]) for j in range(len(rows))])

# normalise the data set
x /= np.linalg.norm(x)
y /= np.linalg.norm(y)

#Plot params
plt.plot(x,y, color = 'b')
plt.xlabel(r'$Distance x(\mu m)$', fontsize = 16)
plt.ylabel('Pixel Intensity (A.U.)', fontsize = 16)
plt.title(r'$\lambda(x;K,{r_e})=1-K*exp(-\frac{x^2}{{r_{e}}^2})$', fontsize = 16)
plt.show()

#Fitting function
def func(x,k,r):
 return 1-k*np.exp(-np.square(x)/np.square(r))

#Curve fitting
fitted_param, fitted_cov = curve_fit(func,x,y)
print(fitted_param)

#Plotting fitted function
plt.plot(x,y, color = 'b')
plt.plot(x,func(x, fitted_param[0], fitted_param[1]), color = 'r')
plt.xlabel(r'$Distance (\mu m)$', fontsize = 16)
plt.ylabel('Pixel Intensity (A.U.)', fontsize = 16)
plt.title(r'$1-K_{fitted}*exp(-\frac{x^2}{{r_{e}^{fitted}}^2})$', fontsize = 16)
plt.legend()
plt.show()
```

For manuscripts utilizing custom algorithms or software that are central to the research but not yet described in published literature, software must be made available to editors and reviewers. We strongly encourage code deposition in a community repository (e.g. GitHub). See the Nature Portfolio guidelines for submitting code & software for further information.

Data

Policy information about availability of data

All manuscripts must include a data availability statement. This statement should provide the following information, where applicable:

- Accession codes, unique identifiers, or web links for publicly available datasets
- A description of any restrictions on data availability
- For clinical datasets or third party data, please ensure that the statement adheres to our policy

All data described in this manuscript are contained within the manuscript, Supplementary Information. Source data are provided in the source data file

Research involving human participants, their data, or biological material

Policy information about studies with human participants or human data. See also policy information about sex, gender (identity/presentation), and sexual orientation and race, ethnicity and racism.

Reporting on sex and gender	NA
Reporting on race, ethnicity, or other socially relevant groupings	NA

Population characteristics	NA
Recruitment	NA
Ethics oversight	NA

Note that full information on the approval of the study protocol must also be provided in the manuscript.

Field-specific reporting

Please select the one below that is the best fit for your research. If you are not sure, read the appropriate sections before making your selection.

☒ Life sciences ☐ Behavioural & social sciences ☐ Ecological, evolutionary & environmental sciences

For a reference copy of the document with all sections, see nature.com/documents/nr-reporting-summary-flat.pdf

Life sciences study design

All studies must disclose on these points even when the disclosure is negative.

Sample size	Based on the published literature and following standard practice of biochemical analysis we consider following sample size for each experiment [Tao, J., Zeng, Y., Dai, B. et al., Nat Commun, 2023(DOI: 0.1038/s41467-023-43826-7), Torosyan, H., Paul, M.D., Forget, A. et al. Nat commun ,2023 (10.1038/s41467-023-38864-0)]. No statistical methods were performed to predetermine sample size.<br>Three biologically independent experiments were performed for the the immunoblot analysis(Figures 2g-h,4b,6g-h and supplementary Figures 1b-c,2g-h,3d-g,4i,7f-h) except Supplementary figure 5c where, two biologically independent experiments were performed. For the single cell imaging experiments, 70-120 cells were imaged from five biologically independent experiments for Figure 2e-f, 3c-d,4e and supplementary figures 2e. Where as 80-100 cells were imaged form six biologically independent experiments for 6b-d, Supplementary Figures 7c. For Supplementary Figure 2c-d, 90-115 cells were imaged from seven biologically independent experiments. For FRAP experiments eight biologically independent experiments were performed for figure 3e, 6e and supplementary figures 4e-g,5a-b. whereas ten independent experiments were performed for figure 4f. All aforementioned experiments were repeated three to ten times with reproducible result each time.
Data exclusions	No data were excluded from the analyses.
Replication	For all immunoblot experiments three biologically independent experiments were performed to confirm the reproducibility. all the immunoblots were successfully reproduced.<br>For determining the diffusion coefficient in each set , Fluorescence Recovery After Photo-bleaching of 20-30 cells were recorded from 8-10 biologically independent experiments to ensure the reproducibility.<br>For single cell assay 70-100 cells were imaged over 4-7 biologically independent experiments.<br>All biochemical and imaging experiments in this paper were repeated independently 3-8 times and all were successfully reproduced.
Randomization	There were no observed heterogeneity in the cell samples used for the experiments. The DNA samples were checked for homogeneity by Sanger sequencing and agarose gel electrophoresis. So, sample allocation is not applicable for our study.
Blinding	Not applicable to our study. Blinding methods are technically not possible for our experiments: Western blot assay, FRAP assay and confocal imaging. No animal experiments were carried out.

Reporting for specific materials, systems and methods

We require information from authors about some types of materials, experimental systems and methods used in many studies. Here, indicate whether each material, system or method listed is relevant to your study. If you are not sure if a list item applies to your research, read the appropriate section before selecting a response.

Materials & experimental systems

n/a	Involved in the study	
☐	☒	Antibodies
☐	☒	Eukaryotic cell lines
☒	☐	Palaeontology and archaeology
☒	☐	Animals and other organisms
☒	☐	Clinical data
☒	☐	Dual use research of concern
☒	☐	Plants

Methods

n/a	Involved in the study	
☒	☐	ChIP-seq
☒	☐	Flow cytometry
☒	☐	MRI-based neuroimaging

Antibodies

Antibodies used	1. VEGFR2 Rabbit monoclonal antibody (Cell SignalingTechnology, Danvers,MA,Cat # 2479S,Lot: 18. 1:1000 for immunobloting)

	2. Phospho-VEGF Receptor 2 (Tyr1175) Rabbit monoclonal antibody (19A10)(Cell Signaling Technology, Danvers, MA, USA. Cat # 2478T Lot:15. 1:1000 for immunobloting and 1:200 for immunofluorescence study)<br>3. VEGFR1 Goat polyclonal antibody (R & D system, Minneapolis, MN, USA) Cat # AF321,Lot: AHT2018011. 1:1000 for immunobloting)<br>4. Human Phospho-VEGFR1/Flt-1 (Y1213) Antibody (R & D system,Minneapolis, MN, USA) Cat # AF4170,Lot: ZKS0318061. 1:1000 for immunobloting and 1:200 for immunofluorescence study)<br>5. Phospho-VEGF Receptor 1 (Tyr1213) Rabbit monoclonal antibody, My BioSource, San Diego, CA, USA) Cat # MBS9600975 ,Lot: 07/2021, 1:200 for immunofluorescence study)<br>6. HA mouse monoclonal antibody (Biolegend,San Diego, CA, USA, Cat # 901501,Lot: B318172.1:2000 for immunobloting)<br>7. Anti phosphotyrosine Antibody (Abcam,Waltham, MA 02453, USA,Cat # ab179530, Lot: GR198792-25. 1:1000 for immunobloting)<br>8. Mouse HRP secondary antibody (Cell Signaling Technology, Danvers,MA, USA Cat # 7076S, Lot: 33. 1:3000 for immunobloting)<br>9. Rabbit HRP Secondary antibody (Abcam,Waltham, MA 02453, USA) Cat# 50095, Lot: 2960660. 1:2000 for immunobloting)<br>10. Goat HRP Secondary antibody (Abcam,Waltham, MA 02453, USA) Cat #Ab6717, Lot: GR267728-27. 1:2000 for immunobloting)<br>11.Rabbit FITC conjugated secondary antibody (Abcam, Waltham, MA 02453, USA) Cat # Ab6885, Lot: GR3391568-I. 1:2000 for immunobloting)<br>See Supplementary Table 1
Validation	1.VEGFR2 Rabbit monoclonal antibody:<br>(A)Specificity / Sensitivity: VEGF Receptor 2 (55B11) Rabbit Monoclonal Antibody detects endogenous levels of VEGF receptor 2 protein. This antibody does not cross-react with other family members. (B)Species Reactivity: Human, Mouse. (C)Application Key: WB-Western Blot IP-Immunoprecipitation IHC-Immunohistochemistry ChIP-Chromatin Immunoprecipitation C&R-CUT&RUN C&T-CUT&Tag DB-Dot Blot eCLIP-eCLIP IF-Immunofluorescence F-Flow Cytometry (D)Citation: Sasset L, Chowdhury KH, Manzo OL, Rubinelli L, Konrad C, Maschek JA, Manfredi G, Holland WL, Di Lorenzo A. Sphingosine-1-phosphate controls endothelial sphingolipid homeostasis via ORMDL. EMBO Rep. 2023 Jan 9;24(1):e54689. doi: 10.15252/embr.202254689. Epub 2022 Nov 21. PMID: 36408842; PMCID: PMC9827560.<br><br>2.Phospho-VEGF Receptor 2 (Tyr1175)<br>(A)Specificity / Sensitivity:<br>Phospho-VEGF Receptor-2 (Tyr1175) (19A10) Rabbit mAb detects endogenous levels of VEGFR-2 proteins only when phosphorylated at tyrosine 1175. This antibody may cross-react with VEGFR1. (B)Species Reactivity: Human, Mouse. (C)Application Key: WB-Western Blot IP-Immunoprecipitation IHC-Immunohistochemistry ChIP-Chromatin Immunoprecipitation C&R-CUT&RUN C&T-CUT&Tag DB-Dot Blot eCLIP-eCLIP IF-Immunofluorescence F-Flow Cytometry. (D)Citation: Boyé K, Geraldo LH, Furtado J, Pibouin-Fragner L, Poulet M, Kim D, Nelson B, Xu Y, Jacob L, Maissa N, Agalliu D, Claesson-Welsh L, Ackerman SL, Eichmann A. Endothelial Unc5B controls blood-brain barrier integrity. Nat Commun. 2022 Mar 4;13(1):1169. doi: 10.1038/s41467-022-28785-9. PMID: 35246514; PMCID: PMC8897508.<br><br>3.VEGFR1 Goat polyclonal antibody<br>(A) Specificity: Detects human VEGFR1/Flt-1 in direct ELISAs and Western blots. immunogen: S. frugiperda insect ovarian cell line Sf 21-derived recombinant human VEGFR1/Flt-1. (B)Species Reactivity: Human. (C) Application: Western Blot , Flow Cytometry , Immunohistochemistry. (D) Citation: Ball SG, Shuttleworth CA, Kielty CM. Vascular endothelial growth factor can signal through platelet-derived growth factor receptors. J Cell Biol. 2007 May 7;177(3):489-500. doi: 10.1083/jcb.200608093. Epub 2007 Apr 30. PMID: 17470632; PMCID: PMC2064818.<br><br>4. Phospho-VEGF Receptor 1 (Tyr1213)(R & D systems)(A) Specificity : Detects human VEGFR1/Flt-1 when phosphorylated at Y1213. (B) Species Reactivity: Human (C) Application: Western Blot (D) Citation:Meda C, Molla F, De Pizzol M, Regano D, Maione F, Capano S, Locati M, Mantovani A, Latini R, Bussolino F, Giraudo E. Semaphorin 4A exerts a proangiogenic effect by enhancing vascular endothelial growth factor-A expression in macrophages. J Immunol. 2012 Apr 15;188(8):4081-92. doi: 10.4049/jimmunol.1101435. Epub 2012 Mar 21. PMID: 22442441.<br><br>5. Phospho-VEGF Receptor 1 (Tyr1213)(My BioSource)<br>(A) Specificity : Phospho-VEGFR1 (Tyr1213) antibody detects endogenous levels of VEGFR1 only when phosphorylated at Tyrosine 1213. (B) Species Reactivity: Human, Mouse, Rat. (C) Application: Western Blot (WB), Immunofluorescence (IF), Immunocytochemistry (ICC), ELISA (EIA). (D) Citation: Davidsson P, Eketjäll S, Eriksson N, Walentinsson A, Becker RC, Cavallin A, Bogstedt A, Collén A, Held C, James S, Siegbahn A, Stewart R, Storey RF, White H, Wallentin L. Cardiovasc Res. 2023 Jul 4;119 (7):1596-1605. doi: 10.1093/cvr/cvad039. PMID: 36869765.<br><br>6. HA mouse monoclonal antibody<br>(A) Specificity: Monoclonal antibody HA.11 was raised against the twelve amino acid peptide CYPYDVPDYASL. (B) Species Reactivity: Mouse. (C) Application: western blot (WB), immunocytochemistry (ICC), immunoprecipitation (IP), and flow cytometry (FC). (D) Citation: Becker, J.R., Clifford, G., Bonnet, C. et al. BARD1 reads H2A lysine 15 ubiquitination to direct homologous recombination. Nature 596, 433–437 (2021). https://doi.org/10.1038/s41586-021-03776-w.<br><br>7. Anti phosphotyrosine antibody<br>(A)Host/specificity: Rabbit monoclonal to Phosphotyrosine. (B)Species Reactivity: Species independent. (C)Application Key: WB-Western Blot, IP-Immunoprecipitation, IF-Immunofluorescence, Dot blot, ELISA. (D)Citation: MO X et al. Elife,2022; Mendelson et al. JCI,2019, Lauenstein JU et al.,JBC,2019.

Eukaryotic cell lines

Policy information about cell lines and Sex and Gender in Research

Cell line source(s)	Chinese Hamster Ovary (CHO) and African green monkey kidney fibroblast-like cell line (Cos-7) cell lines were purchased from National Centre for Cell Science Cell Repository - Pune. Murine macrophage (RAW264.7) cell line was purchased from

	National Centre for Cell Science Cell Repository - Pune and ATCC.
Authentication	None of the cell line used were authenticated
Mycoplasma contamination	CHO and COS-7 cell lines were tested mycoplasma negative. Mycoplasma test was done by 16S rRNA PCR based method. The primers used for mycoplasma detection are given below:<br>Myco-FP GGCGAATGGGTGAGTAACACG<br>Myco-RP CGGATAACGCTTGCGACCTATG<br><br>RAW264.7 cell line was not tested for mycoplasma contamination.
Commonly misidentified lines<br>(See ICLAC register)	No commonly misidentified lines were used in the study
